# Changes in gray matter volume after microsurgical lumbar discectomy: a longitudinal analysis

**DOI:** 10.3389/fnhum.2015.00012

**Published:** 2015-02-05

**Authors:** Michael Luchtmann, Sebastian Baecke, Yvonne Steinecke, Johannes Bernarding, Claus Tempelmann, Patrick Ragert, Raimund Firsching

**Affiliations:** ^1^Department of Neurosurgery, Otto-von-Guericke-University MagdeburgMagdeburg, Germany; ^2^Institute of Biometry and Medical Informatics, Otto-von-Guericke-University MagdeburgMagdeburg, Germany; ^3^Department of Neurology, Otto-von-Guericke-University MagdeburgMagdeburg, Germany; ^4^Department of Neurology, Max Planck Institute for Human Cognitive and Brain SciencesLeipzig, Germany

**Keywords:** chronic low back pain, lumbar disc herniation, voxel-based morphometry, structural brain alterations, gray matter volume, microsurgical lumbar discectomy

## Abstract

People around the world suffer chronic lower back pain. Because spine imaging often does not explain the degree of perceived pain reported by patients, the role of the processing of nociceptor signals in the brain as the basis of pain perception is gaining increased attention. Modern neuroimaging techniques (including functional and morphometric methods) have produced results that suggest which brain areas may play a crucial role in the perception of acute and chronic pain. In this study, we examined 12 patients with chronic low back pain and sciatica, both resulting from lumbar disc herniation. Structural magnetic resonance imaging (MRI) of the brain was performed 1 day prior to and about 4 weeks after microsurgical lumbar discectomy. The subsequent MRI revealed an increase in gray matter volume in the basal ganglia but a decrease in volume in the hippocampus, which suggests the complexity of the network that involves movement, pain processing, and aspects of memory. Interestingly, volume changes in the hippocampus were significantly correlated to preoperative pain intensity but not to the duration of chronic pain. Mapping structural changes of the brain that result from lumbar disc herniation has the potential to enhance our understanding of the neuropathology of chronic low back pain and sciatica and therefore may help to optimize the decisions we make about conservative and surgical treatments in the future. The possibility of illuminating more of the details of central pain processing in lumbar disc herniation, as well as the accompanying personal and economic impact of pain relief worldwide, calls for future large-scale clinical studies.

## Introduction

The brain has a remarkable capacity for neuroplastic changes that occur during the process of learning from and adapting to an altered environment (Markham and Greenough, 2004; Adkins et al., 2006; Draganski and May, 2008). Such changes take place on the molecular, functional, and structural levels. In recent years researchers have studied a wide range of pain disorders using brain imaging techniques like voxel-based morphometry and have discovered pain-related structural alterations of the central nervous system (CNS) that indicate that functional as well as structural changes of the CNS play a crucial role in mediating acute and chronic pain disorders (May, 2011). While acute pain is associated with structural changes occurring predominantly in the somatosensory system, particularly in the thalamus (Apkarian et al., 2005; Decharms et al., 2005), chronic pain is believed to be more complex (May, 2008, 2011). The precise locations of these pain-related areas vary considerably across the conducted studies. Nevertheless, changes in gray matter volume were frequently observed in the frontal gyrus, basal ganglia, insula, thalamus, anterior cingulate cortex, and hippocampus, giving rise to the concept of the *pain matrix* (Smallwood et al., 2013a).

Lumbar disc herniation (LDH), a common cause of chronic pain like sciatica and low back pain (LBP) disables millions of people and generates tremendous physical and economic strain (Dagenais et al., 2008; Wenig et al., 2009). Sciatica is defined as pain radiating into at least one leg in a classic dermatomal distribution. It is usually mediated by a single nerve root in the lumbosacral spine and may be accompanied by motor weaknesses or sensory deficits. Using a cross-sectional approach, we observed in our recent study (Ashburner and Friston, 2000) (which used the same participants—patient and control—that were used in this study) several cortical and subcortical regions with altered gray matter volume in the patients with chronic low back pain due to LDH (Luchtmann et al., 2014). These changes were visible in several brain areas believed to be involved in pain processing, including the basal ganglia, the anterior cingulate cortex, and the frontal cortex. It may well be possible that understanding these changes of the brain that result from chronic pain due to LDH has the potential to enhance our understanding of the neuropathology of chronic LBP and sciatica and therefore may help to optimize future treatment. In the present exploratory study, therefore, we investigated the gray matter volume in the brains of patients with chronic back pain and sciatica resulting from LDH both before and after they had undergone a lumbar discectomy. We hypothesized that regions associated with the pain matrix would show significant changes in the volume of the gray matter post-discectomy, and that the alterations found in our recently published study would be at least partially reversible after successful pain relief from the microsurgical lumbar discectomy.

## Patients and methods

The ethics committee of the Medical Faculty of the University of Magdeburg approved the study in compliance with national legislation and the Code of Ethical Principles for Medical Research Involving Human Subjects of the World Medical Association (Declaration of Helsinki).

### Subjects

Twenty-four right-handed subjects were enrolled in the study and were divided into two groups. The first group consisted of 12 LDH patients (mean age 43.9 ± 12.9 years, 6 female and 6 male) with LBP and sciatica. Each patient had been experiencing chronic pain for at least 3 months (mean pain duration: 9.3 ± 5.4 month). All patients were diagnosed, using spinal MRI, with an isolated LDH at either level L4-5 or L5-S1. Half of these patients suffered from sciatica located in the left lower limb and half from right-sided sciatica. The spinal MRI was assessed using the usual clinical routine by experienced neurosurgeons and neuroradiologists. The second, or control, group consisted of 12 subjects age and gender matched to the first group. The members of this control group had no history of any chronic pain or neurological disorders. All participants gave their informed written consent. Prior to the MR imaging, patients and control-group volunteers were examined neurologically. The initial mean level of back pain was quantified using a common visual analog scale (VAS) ranging from 0 to 10 points, where 0 points indicates no pain. The control group reported a mean pain intensity of 0 and the neurological examination revealed no neurological deficits. Any changes in patients' VAS scores were assessed using a paired *t*-test with an alpha-level of 5% for statistical significance.

### Experimental procedure

A high-resolution MRI image of each patient's brain was acquired 1 day prior to his or her scheduled microsurgical lumbar discectomy. All patients underwent a complication-free surgery on the following day, at either level L4-5 or L5-S1. A second MRI was taken 4 weeks after the patient's discharge from the hospital (mean time between both imaging sessions 36.8 ± 3.1 days). The subjects from the control group were also scanned twice, with a temporal gap of 38.0 ± 3.9 days between the two MRI sessions. No member of the control group underwent any intervening medical procedures.

### MR imaging

MR imaging was conducted on a 3 Tesla Siemens Magnetom Trio scanner (Erlangen, Germany) using an 8-channel phased-array head coil. A high-resolution anatomical dataset of the entire brain was acquired using a T1-weighted, magnetization-prepared rapid gradient-echo (MPRAGE) sequence (field of view = 256 mm, matrix size = 256 × 256, slices = 192, slice thickness = 1 mm, repetition time = 2500 ms, flip angle = 7%) resulting in an isotropic voxel resolution of 1 mm^3^.

### MRI data preprocessing and analysis

The anatomical high-resolution images were analyzed using the voxel-based morphometry toolbox [VBM8 (Ashburner and Friston, 2000)] implemented in SPM8, which runs under Matlab. The image data of all patients and volunteers were bias corrected, segmented, and registered to the standardized Montreal Neurological Institute (MNI) space using the segmentation approach developed by Ashburner and Friston (2005). Gray matter (GM) was scaled using the Jacobian determinants of the deformation to account for distortions during linear and non-linear transformation (Ashburner and Friston, 2005). Subsequently, the modulated GM densities were smoothed by an 8 mm FWHM (full-width at half-maximum) Gaussian kernel. For the statistical analysis, we excluded all voxels with a GM value below 0.2 to minimize partial-volume effects near the border between gray and white matter. GM differences within each group were estimated with a flexible factorial design using subjects and time as factors. The individual pain level (VAS) at the time of MRI (before and after surgery) was included as a covariate in the design matrix to seek brain regions whose GMV alterations appears to be related to the change of the pain after surgery. Results were family-wise error corrected (FWE, *p* = 0.05) for multiple comparisons in the context of Gaussian random field theory (Friston et al., 1996). Additionally, a non-stationary cluster extent correction (FWE, *p* = 0.05) implemented in VBM8 was applied (Hayasaka et al., 2004). All data and images are displayed in accord with neurological convention.

Additionally, to assess the relevance of preoperative pain intensity and duration and the impact of postoperative changes of the pain to the structural alterations found in the VBM analysis, we performed regression analyses (linear regression model, ANOVA) using preoperative VAS scores, pain duration and the postoperative pain alteration as independent variables in an additional step in order to predict the changes in GM volume (dependent variable). For this purpose the corresponding voxels were extracted using the toolbox Marsbar (Version 0.42, http://marsbar.sourceforge.net/). The subsequent analyses were conducted with SPSS (Version 21, http://www.ibm.com/software/de/analytics/spss/).

## Results

LDH patients reported a preoperative mean pain intensity level of 7.2 ± 0.9, ranging between 6 and 9. All of these patients suffered from LBP and unilateral sciatica that radiated into at least one leg in a classic dermatomal distribution (The mean preoperative pain duration was 37 ± 21.7 weeks). For each of them, the pain had been accompanied by numbness and tingling for at least 3 months. The clinical examination revealed no motor deficit in any patient. The MRI examination of the spine discovered LDH at the level of L4-5 in five patients and at the level of L5-S1 in seven patients. All operations were performed without any ensuing complications. The postoperative VAS score at the time of the second MRI examination was significantly lower (*p* < 0.001) than the preoperative score, with a mean value of 1.8 ± 1.1, ranging between 0 and 4.

### Analyses of gray matter changes

**Figure 1:**
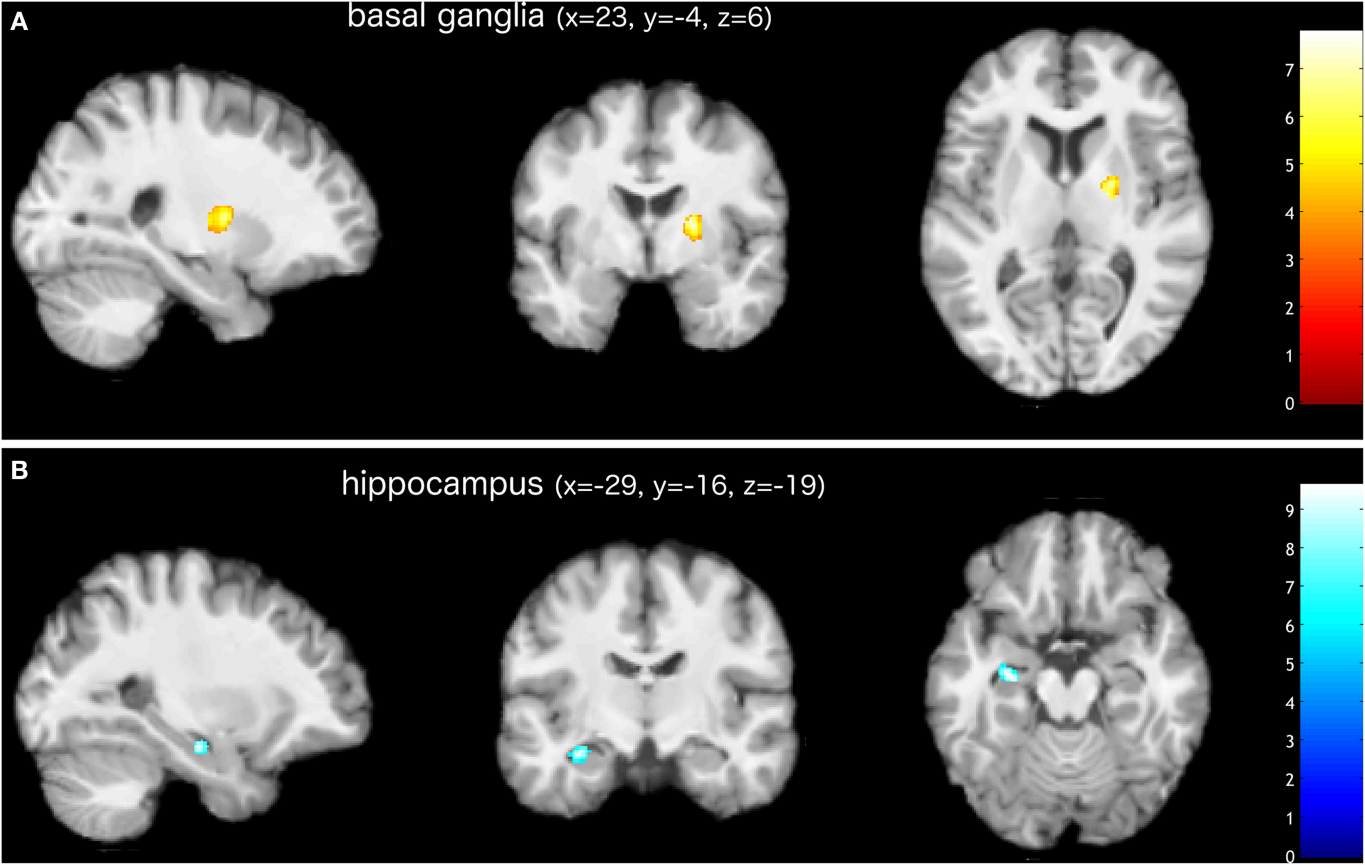
**Increased (A) and decreased (B) gray matter volume in patients after surgical treatment of lumbar disc herniation (corrected for multiple comparisons, FWE *p* < 0.05)**. The group analysis of LDH patients showed increased gray matter volume in the right basal ganglia (putamen and pallidum). Decreased gray matter volume was found in the left hippocampus. Images are presented in neurological convention. Bars indicate *t*-values.

**Figure 2:**
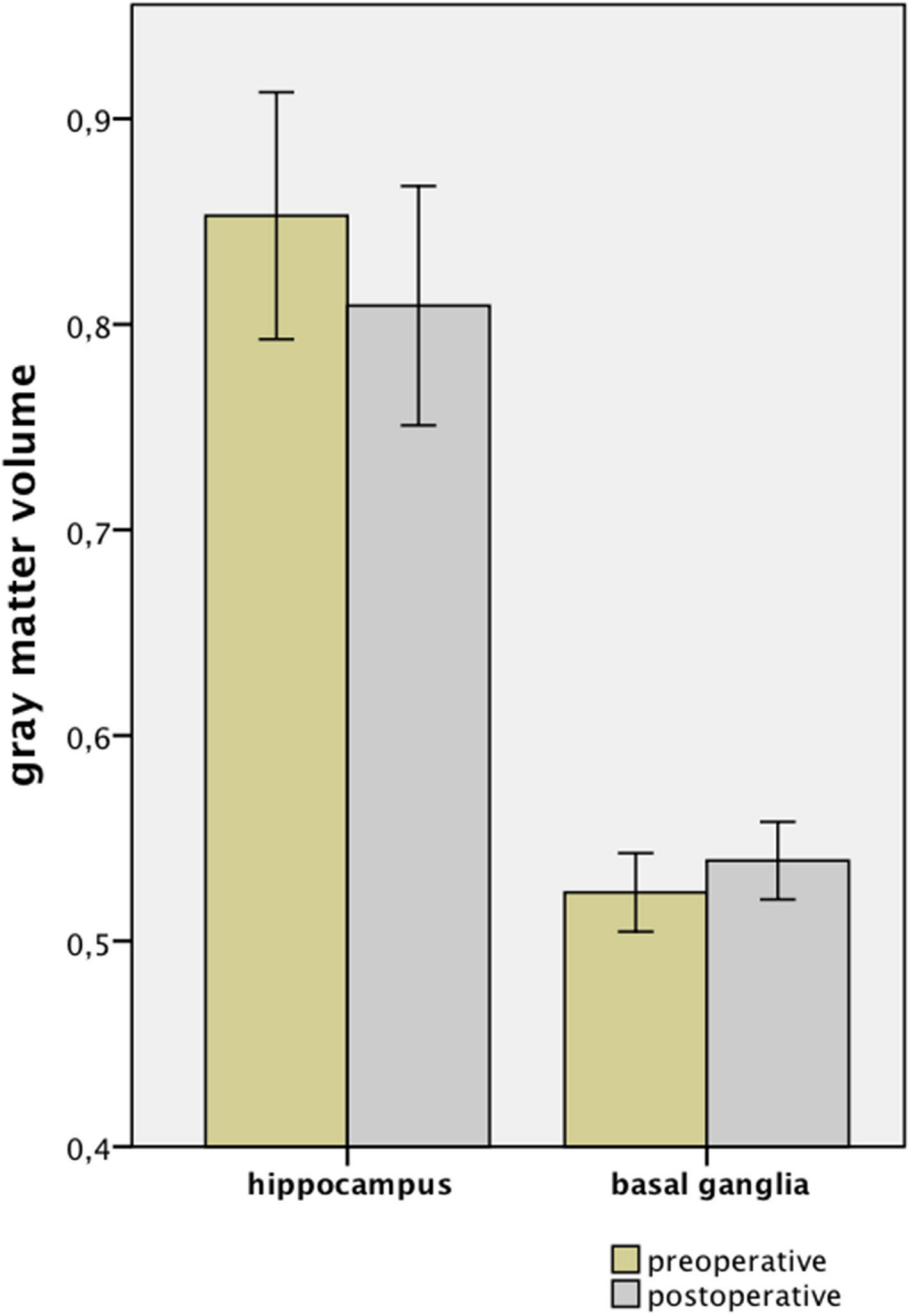
**Absolute GM volume alterations after lumbar discectomy**.

The examination of the T1-weighted MRI data of all 24 subjects revealed no pathological brain alterations. Figure 1 and Table 1 show the GM alterations of the group-level analysis of the LDH patients; the group-level analysis of the control group showed no significant GM changes of the brain. Compared to the images taken prior to the surgical treatment, these latter images of LDH patients showed significantly reduced GM volume in the left hippocampus after lumbar discectomy. A postoperative increase in GM volume, however, was found in the right pallidum and putamen, as part of the basal ganglia (see Figure 2). None of these changes appears to be significantly related to the altered pain intensity (covariate).

**Table 1:** **Significant gray matter changes in LDH patients after surgical treatment**.

**Region**	**Hemisphere**	**GM**	**MNI coordinates in mm**			**Z-score of peak-change**	**Cluster size *k* = 1 mm^3^**
			***x***	***y***	***z***		
Hippocampus	Left	Decrease	−29	−16	−19	4.87	89
Basal ganglia	Right	Increase	23	−4	6	4.44	222

*(Corrected for multiple comparisons, FWE p < 0.05)*.

### Regression analysis

Table 2 shows the results of the linear regression analyses. These results reveal a weak but significant influence of pain intensity (VAS) prior to the surgical treatment on the GM alterations observed after surgery (the higher the pain the higher GM alteration). In the present data, that influence is limited to the hippocampus. The preoperative duration of pain and the postoperative pain alteration, however, seems to have had no linear influence on any subsequent GM alterations.

**Table 2:** **Results of the regression analysis used to estimate the influence of preoperative pain on the gray matter alterations occurring after surgical treatment of lumbar disc herniation**.

**Independent variable**	**Dependent variable GM alteration *Region***	**Regression coefficient beta**	***p*-value**
***Pain***			
Preoperative duration	Hippocampus	0.019	0.385
Preoperative duration	Basal ganglia	0.021	0.284
Preoperative intensity	Hippocampus	0.180	**<0.001**
Preoperative intensity	Basal ganglia	0.009	0.666
Postoperative change	Hippocampus	0.109	0.736
Postoperative change	Basal ganglia	−0.387	0.214

*(Significant results are highlighted in bold)*.

## Discussion

In the present study, using voxel-based morphometry (VBM), we observed a significant alteration in the GM volume of patients who had undergone surgical treatment to relieve chronic low back pain (cLBP) and sciatica resulting from lumbar disc herniation (LDH). After correcting for multiple comparisons, we found in these patients a region within the hippocampus where the GM had decreased in volume after surgery. We observed an increase in GM volume, however, in the putamen and pallidum, parts of the basal ganglia (Figures 1, 2 and Table 1). The regression analysis revealed evidence of a slight but significant influence of preoperative pain intensity (VAS) on the observed alterations in GM volume in the hippocampus. The duration of the preoperative pain, however, seems to have had no linear influence on any changes in GM volume.

In recent years, a large body of evidence has revealed structural brain alterations associated with several chronic pain syndromes (May, 2008, 2011; Smallwood et al., 2013a) similar to those suffered by patients in our study. Smallwood et al. (2013a) conducted quantitative meta-analyses of studies that revealed structural differences between the brains of individuals with chronic pain, including cLBP, and the brains of individuals in healthy control groups. In those studies, the only regions in the brains of the chronic pain sufferers that were found to have increased in GM volume were the hippocampus and the parahippocampal gyrus. It is interesting to note that, in contrast to those results, we in fact found a decrease in GM volume within the left hippocampal formation after surgical treatment. The reverse constellation was found around the region of the basal ganglia (BG). While Smallwood et al. (2013a) observed in their meta-analyses that the largest, most significant decrease in GM volume of chronic pain patients was in a region including the putamen and the claustrum, the results of the present study show the largest increase in GM volume in the pallidum and putamen, indicating that GM alteration as found in the hippocampus and the BG is potentially reversible after successful surgical treatment of the underlying cause of cLBP and sciatica. However, since the sample size is too small for reliable longitudinal analysis between the patients and the control group, we cannot definitively eliminate the possibility that the observed GM volume alterations represent a further deviation away from normal morphometry.

The hippocampus and the BG are part of the contemporary model of the pain matrix and, as such, are believed to be crucially involved in pain processing and modulation. Recent evidence suggests that the BG are uniquely involved in the thalamo-cortico-BG loops that integrate the motor, emotional, autonomic, and cognitive aspects of chronic pain (Borsook et al., 2010). Specific pain-induced activations in the caudate nucleus have been proposed to be a central part of the pain modulatory system of the brain. Some researchers believe that these activations may reduce the affective components of pain and lead to its disrupted processing. Subcortical structural changes in these regions may therefore be associated with uncontrollable chronic pain (Borsook et al., 2010). We recently observed several cortical and subcortical regions with alterations in GM volume in patients with cLBP due to LDH, including a reduction in the GM volume in the BG (Luchtmann et al., 2014). Smallwood et al. (2013a) likewise found these reductions in GM volume. In contrast, our present study revealed a significant increase in the GM volume in the BG after successful surgical treatment. The precise function of the BG in pain processing and modulation is not yet fully understood. Nevertheless, an increase in GM volume is consistent with the sustained pain relief and perceived recovery that occur when restored feed-forward and feedback loops lead to a recovered undisturbed affectivity. The hippocampus is critically involved in anxiety, learning, and memory, and it is essential in contextual conditioning and extinction (Phillips and Ledoux, 1992). Moreover, it is considered the primary brain structure for storage and retrieval of long-term explicit memories. Until recently, it was thought that the hippocampus was not essential to the process of pain modulation, but an increasing number of experimental studies have confirmed that the hippocampus receives afferent pain impulses and plays an important role in pain modulation (Wang, 2008). Mutso et al. (2012) hypothesized that chronic pain can be redefined in terms of context conditioning and extinction, and that it can be viewed as a state of continual learning through which aversive emotional associations are constantly made with otherwise incidental events precisely because of the persistent presence of a chronic pain. In their study, Mutso et al. (2012) revealed multiple hippocampal abnormalities in both chronic pain patients and animals and demonstrated a potential underlying mechanism for hippocampal-mediated behavioral abnormalities through an altered short-term synaptic plasticity. A reduced GMV in the hippocampal formation, as found in our study, might be a correlate of perceived recovery that occurs after successful treatment of the underlying cause of chronic pain. The hippocampus has a decisive role in anxiety processing (Ploghaus et al., 2001; Mutso et al., 2012). Hsu et al. (2009) showed that the GMV in the left insula is inversely correlated with trait anxiety, indicating that specific structural alterations could be attributed to affective disturbances. Since no trait anxiety scores were gathered in the present study, we cannot fully exclude it as a possible confounding factor in the observed regions.

The sample size and limited follow-up data on outcomes for controls warrants careful interpretation of the results. However, the study itself provides additional evidence that chronic lumbar pain and its relief may both produce GM alterations. There are only a few studies thus far that have examined GM alterations after successful surgical treatment, particularly with regard to treatment for cLBP. In one previous study, Gwilym et al. (2010) showed that chronic pain and disability in patients with hip osteoarthritis might lead to decreases of GMV within the thalamus. Their results also show that the thalamic volume changes reverse after hip arthroplasty, and that these reversals are associated with decreased pain and increased function. The authors emphasize potential implications with regard to optimizing the timing of orthopedic interventions such as arthroplasty. In another study, Rodriguez-Raecke et al. (2013) similarly found changes in brain GM in patients in the chronic pain state with primary hip osteoarthritis that partially reversed when the patients became pain free following hip joint endoprosthetic surgery. An increase in GM volume after those successful surgeries was found in the same areas where a decrease in GM volume had been observed prior to the surgery. The authors appropriately indicate that structural brain alterations in chronic pain patients probably reflect neither neuronal damage nor atrophy. Furthermore, Seminowicz et al. (2011) demonstrated that the left dorsolateral prefrontal cortex (DLPFC), which was thinner and presented abnormal cognitive task-related activity in chronic-low-back-pain patients before treatment as compared to the DLPFC of the control group, became significantly thicker and presented normal activity following treatment. In contrast to these results, we found no GMV alterations within or near the DLPFC. This lack of consistency is difficult to explain, but may be because different underlying neurophysiological processes lead to pain chronification. Regarding the multifactorial etiologies of chronic pain syndromes, another previous study indicated that the impact of cLBP on structural as well as on functional brain alterations may vary according to the type of pain involved (neuropathic vs. non-neuropathic) (Apkarian et al., 2004). We suggest that a distinction in the specific etiologies and treatments is necessary in order to assess structural and functional brain alterations.

Our study does align with other reports where no GMV alterations were found in the primary somatosensory cortex. While short-lived pain leads to a substantial increase in the GM volume of the somatosensory cortex (Teutsch et al., 2008), no changes in these regions were found in patients suffering from chronic pain. As it stands, why some people develop chronic pain syndromes is unknown. It seems that chronic pain patients have perhaps lost the ability to habituate to pain (Peters et al., 1989; Flor et al., 2004). Some researchers explain the absence of GM volume changes in the somatosensory cortex as stemming from cognitive maladaptation to acute pain. They speculate that significant harmful input is no longer present and that it is the brain that mostly drives the experience of a constant pain (Teutsch et al., 2008). Using longitudinal brain imaging, Baliki et al. (2012) recently reported that structural as well as functional alterations in the brain reliably distinguished between those subjects with subacute back pain who subsequently recovered within a year and those for whom the pain persisted after that time period. The present results support the theory that a timely intervention may prevent patients from developing such cerebral maladaptation, restore normal brain function and structure, and lead to sustained recovery. Most guidelines suggest that, in order to avoid chronification of pain, surgical intervention should occur after 6 weeks of non-operative treatment for patients with persistent symptomatic LDH whose symptoms are severe enough to warrant surgery (Schoenfeld and Weiner, 2010; Kreiner et al., 2014). Interestingly, our results suggest that the duration of pain prior to the surgical treatment has less influence (positive correlation) on the observed GMV alterations than the intensity of the pain. This fact underlies the complexity of the pathophysiological processes that lead, in the end, to chronification of pain.

Some limitations of this study should be addressed. The sample size was relatively small, but the results are significant after correction for multiple comparisons (*p* < 0.05, FWE corrected). Also, the VBM analysis cannot account for the underlying cytoarchitectonic causes of the structural alterations of the CNS. Variable cell sizes, increased or decreased synaptogenesis, or a changed quantity of glial cells might account for the structural alterations found (Schmidt-Wilcke et al., 2007). Furthermore, all patients took painkillers, which were discontinued after surgery. Since many LDH patients use opioid analgesic substances for the treatment of pain, the idea that chronic opioid exposure might lead to a dose-dependent GM volume increase in the cingulate cortex (Younger et al., 2011) is intriguing. Despite the different anatomical regions involved with opioid exposure and despite the fact that only one of the 12 patients involved in this study took a low-potency opioid for a few days, medication cannot be fully excluded as a confounding factor. It should also be taken into account that behavioral changes resulting from the reduction or absence of cLBP (such as enhancement of agility, engaging in physical training, and making other lifestyle changes) and/or the natural course of the condition may also contribute to the GM volume alterations that were observed after microsurgical lumbar discectomy.

## Conclusion

We conclude that chronic pain due to LDH can induce structural alterations of the brain that are potentially reversible after successful discectomy. Since pain is ultimately interpreted by the brain, studying the cortical and subcortical changes of the brain that result from LDH has the potential to enhance our understanding of the neuropathology of chronic LBP and sciatica and therefore may help to optimize future conservative and surgical treatment options. Specifically, the classification of specific morphometric changes of the brain associated with the treatment of LDH may possibly prove useful in identifying individual predictive factors in the evaluation of whether a patient is likely to improve by non-surgical management or would instead benefit from surgery. It is interesting to speculate whether more advanced neuroimaging techniques and data-processing methods would enable the identification of specific patterns of altered brain functions and anatomy and thus yield additional objective diagnostic criteria that might then guide therapeutic interventions targeting the brain for effective individual management of LDH. The ability to illuminate and understand more of the details underlying central pain processing in LDH, and the potential economic impact of such an amplified understanding, to our mind justifies future large-scale clinical studies conducted to confirm or modify the results that we have presented here.

### Conflict of interest statement

The authors declare that the research was conducted in the absence of any commercial or financial relationships that could be construed as a potential conflict of interest.
